# Analysis of Gut Microbiota in Rheumatoid Arthritis Patients: Disease-Related Dysbiosis and Modifications Induced by Etanercept

**DOI:** 10.3390/ijms19102938

**Published:** 2018-09-27

**Authors:** Andrea Picchianti-Diamanti, Concetta Panebianco, Simonetta Salemi, Maria Laura Sorgi, Roberta Di Rosa, Alessandro Tropea, Mayla Sgrulletti, Gerardo Salerno, Fulvia Terracciano, Raffaele D’Amelio, Bruno Laganà, Valerio Pazienza

**Affiliations:** 1 Department of Clinical and Molecular Medicine, Sant’Andrea University Hospital, Sapienza University of Rome, 00185 Rome, Italy; s.salemi@gmail.com (S.S.); marialaura.sorgi@uniroma1.it (M.L.S.); roberta.dirosa@uniroma1.it (R.D.R.); alessandro.tropea3@gmail.com (A.T.); Mayla.sgrulletti@uniroma1.it (M.S.); Gerardo.salerno@uniroma1.it (G.S.); raffaele.damelio@uniroma1.it (R.D.); bruno.lagana@uniroma1.it (B.L.); 2 Gastroenterology Unit, IRCCS “Casa Sollievo della Sofferenza” Hospital, Viale dei Cappuccini, 1, 71013 San Giovanni Rotondo, Italy; panebianco.c@gmail.com (C.P.); terracciano74@hotmail.com (F.T.)

**Keywords:** microbiota, rheumatoid arthritis, anti-TNF-α, methotrexate, etanercept, disease activity

## Abstract

A certain number of studies were carried out to address the question of how dysbiosis could affect the onset and development of rheumatoid arthritis (RA), but little is known about the reciprocal influence between microbiota composition and immunosuppressive drugs, and how this interaction may have an impact on the clinical outcome. The aim of this study was to characterize the intestinal microbiota in a groups of RA patients treatment-naïve, under methotrexate, and/or etanercept (ETN). Correlations between the gut microbiota composition and validated immunological and clinical parameters of disease activity were also evaluated. In the current study, a 16S analysis was employed to explore the gut microbiota of 42 patients affected by RA and 10 healthy controls. Disease activity score on 28 joints (DAS-28), erythrocyte sedimentation rate, C-reactive protein, rheumatoid factor, anti-cyclic citrullinated peptides, and dietary and smoking habits were assessed. The composition of the gut microbiota in RA patients free of therapy is characterized by several abnormalities compared to healthy controls. Gut dysbiosis in RA patients is associated with different serological and clinical parameters; in particular, the phylum of Euryarchaeota was directly correlated to DAS and emerged as an independent risk factor. Patients under treatment with ETN present a partial restoration of a beneficial microbiota. The results of our study confirm that gut dysbiosis is a hallmark of the disease, and shows, for the first time, that the anti-tumor necrosis factor alpha (TNF-α) ETN is able to modify microbial communities, at least partially restoring a beneficial microbiota.

## 1. Introduction

The human intestinal microbiota is a complex microcosm composed of more than 1000 different bacterial species, archaea, fungi, and viruses [1]. There is growing knowledge that these bacteria are not only involved in the digestion and absorption of food, but they can also exert a protective function by preventing adherence of pathogenic bacteria to the mucosal layer, and they play a pivotal role in modulating the innate and acquired immunity of the host [2,3,4].

Recent advances in sequencing technologies led to a deep characterization of the human gut microbiota in healthy subjects. This enabled the investigation of modifications in the structure of gut commensal communities (called dysbiosis), which could be involved in the onset and maintenance of different chronic autoimmune diseases, such as inflammatory bowel diseases (IBD) and arthritis [5,6]. Dysbiosis could lead to alterations in the intestinal epithelial cell layer with an increased exposure to a variety of bacteria and bacterial products leading to a chronic antigenic stimulation, spreading of inflammatory mediators, and T cell activation [7,8]. 

It was recently demonstrated that different environmental factors are involved in the development of both intestinal/oral dysbiosis and arthritis onset and outcome, among which the most relevant are diet, smoking, infections, and drugs [9,10,11,12].

Rheumatoid arthritis (RA) is an inflammatory autoimmune disease of unknown etiology, potentially leading to progressive joint destruction and disability. In RA patients, an accumulating body of studies demonstrated a pathogenic role of dysbiosis of the oral microbiota, in particular, an association between *Porphyromonas gingivalis*, periodontitis, and the generation of citrullinated products was clearly demonstrated [13,14,15]. 

On the other hand, data regarding the role of intestinal microbiota in these patients are not conclusive, and no studies address the reciprocal influence between biotechnological immunosuppressants and microbiota on RA outcome.

Etanercept (ETN, a dimeric recombinant fully human fusion protein consisting of a human 75-kDa tumor necrosis factor (TNF) receptor linked to the Fc portion of human immunoglobulin G1 (IgG1)) is one of the five currently available biotechnological agents that target TNF-α. ETN proved to be safe and effective in reducing disease activity and limiting the progression of joint damage in RA patients; it can be administered as monotherapy or in combination therapy with methotrexate (MTX) [16].

The aim of this study was to characterize the intestinal microbiota in a group of RA patients treatment-naïve, under MTX, and/or ETN. Correlations between the gut microbiota composition and validated immunological and clinical parameters of disease activity were also evaluated.

## 2. Results

### 2.1. Microbiota Profile in Rheumatoid Arthritis (RA) Patients Free of Therapy versus Healthy Controls(HCs)

Lifestyle factors, as well as demographic, serologic, and clinical parameters, of the four RA treatment groups are shown in Table 1. 

No significant differences were observed among the RA groups except for the disease duration, which, as expected, was shorter in the group of naïve and MTX monotherapy patients. HCs were also similar to RA patients regarding lifestyle factors, but they were significantly younger (*p* < 0.05). 

**Figure 1:**
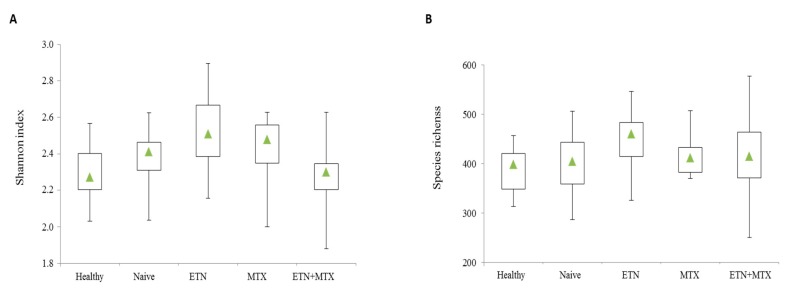
Box plots of Shannon diversity index (**A**) and species richness (**B**) of microbiota of healthy controls and different treatment groups of rheumatoid arthritis (RA) patients. The triangle represents the median value.

After passing quality control filters, a mean of 281,218 sequences per sample were obtained. The assessment of the Shannon index in each sample revealed that α-diversity was neither changed in naïve patients compared with the HCs, nor in each treatment group (naïve, ETN, MTX, or ETN plus MTX) relative to patients free of therapy (Figure 1A). Similarly, no significant change in species richness was observed (Figure 1B).

**Figure 2:**
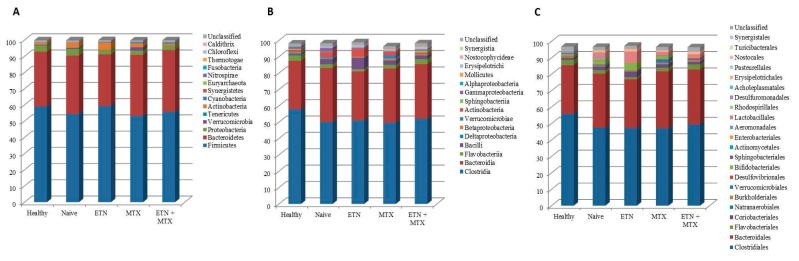
Microbiota composition of healthy controls and different treatment groups of RA patients at the phylum (**A**), class (**B**), and order (**C**) levels. The mean value of the eight top taxonomic classifications at each level is represented.

**Figure 3:**
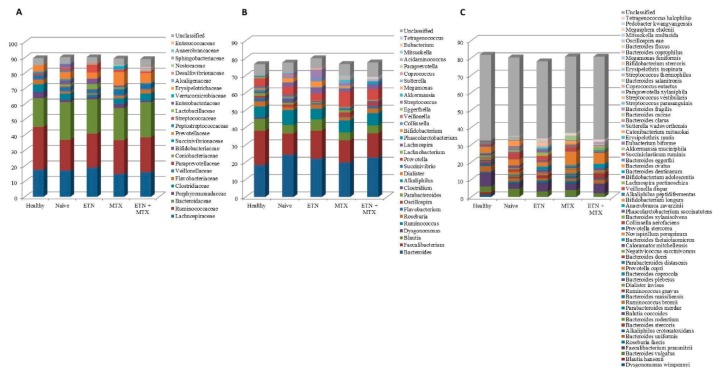
Microbiota composition of healthy controls and different treatment groups of RA patients at the family (**A**), genus (**B**), and species (**C**) levels. The mean value of the eight top taxonomic classifications at each level is represented.

As a first approach, we compared the gut microbiota composition of the HCs with that of RA patients free of therapy. This analysis revealed that the relative abundance of the microbial phyla was almost unchanged (Figure 2A), while significant differences between the two groups were found at lower taxonomic levels. The most striking alterations were a five-fold increase in the class of Bacilli (Figure 2B) and a seventeen-fold increase in the order of Lactobacillales (Figure 2C) found in RA patients with respect to the controls (2.89 ± 3.19 vs. 0.58 ± 0.39; *p* = 0.035, and 2.69 ± 3.15 vs. 0.15 ± 0.34; *p* = 0.021, respectively). Significant reductions of the genus *Faecalibacterium* (12.21 ± 7.4 vs. 19.72 ± 4.41; *p* = 0.012) (Figure 3B) and its cognate species *Faecalibacterium prausnitzii* (3.23 ± 3.22 vs. 7.97 ± 3.78; *p* = 0.006) (Figure 3C) were also found. Furthermore, significant changes were also observed in the genus *Flavobacterium* (Figure 3B) and the species *Blautia coccoides* (Figure 3C), which were both represented in the control group, but were not detected at all in RA naïve patients (2 ± 2.44 vs. 0 ± 0; *p* = 0.013, and 0.7 ± 0.76 vs. 0 ± 0; *p* = 0.006).

### 2.2. Microbiota Profile in RA Patients Free of Therapy versus Treated Patients

We next sought to evaluate any difference in microbiota composition of RA patients based on whether they were free of therapy or they received ETN, MTX, or a combination of the two. Mounting evidence supports the existence of a reciprocal connection between drugs and microbiota, which can influence each other and have an impact on therapeutic outcomes [17]. Specifically, MTX was shown to modify microbiota composition, partly restoring the microbial balance altered by the disease [12]. 

When compared to naïve patients, major changes were observed in the ETN group. The Cyanobacteria significantly increased (0.49 ± 0.5 vs. 0.08 ± 0.07; *p* = 0.016) (Figure 2A), and the same increase was observed in the Nostocophycideae class (Figure 2B) and the Nostocales order (0.35 ± 0.5 vs. 0 ± 0; *p* = 0.031) (Figure 2C), which both belong to the phylum of Cyanobacteria. In detail, Nostocophycideae and Nostocales, which were not represented among the naïve subjects, were instead detected in four out of ten ETN patients. In addition, the class of Deltaproteobacteria (0.07 ± 0.23 vs. 0.57 ± 0.72; *p* = 0.05) (Figure 2B) and the family of Clostridiaceae (1.51 ± 1.76 vs. 3.97 ± 3.4; *p* = 0.05) (Figure 3A) significantly decreased in the ETN group as compared to the naïve. The only statistically significant alteration found in the MTX group was a decrease in the relative abundance of Enterobateriales (0.07 ± 0.24 vs. 0.85 ± 1.22; *p* = 0.05) (Figure 2C), while no significant changes were observed in RA patients upon ETN plus MTX therapy.

### 2.3. Association of Microbiota Profile with Clinical Pathological Features in RA Patients

**Figure 4:**
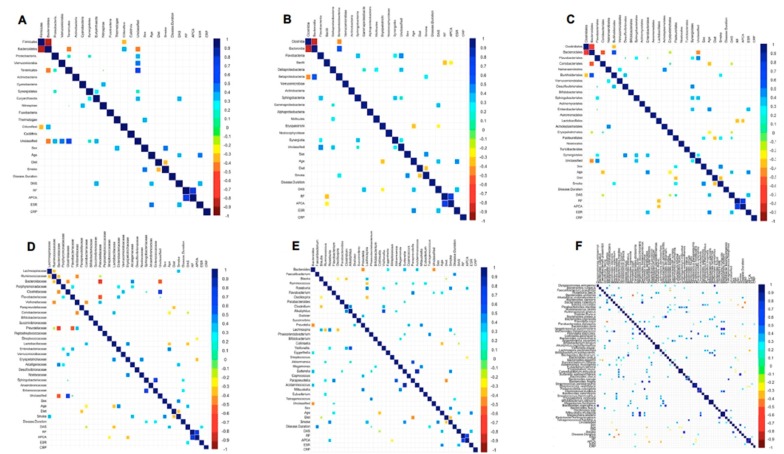
Association of gut microbiota profile with clinical pathological features in RA patients at the phylum (**A**), class (**B**), order (**C**), family (**D**), genus (**E**), and species (**F**) levels.

Finally, we wondered whether microbiota composition could be associated to clinical parameters (i.e., sex, age, disease duration, disease activity Score on 28 joints (DAS-28), rheumatoid factor (RF), anti-cyclic citrullinated peptides antibodies (ACPA), erythrocyte sedimentation rate (ESR) and C-reactive protein (CRP) and lifestyle factors (diet and smoking habits) of the RA patients. For this purpose, correlations were analyzed between microorganisms at each taxonomic level and all the above described parameters (Figure 4).

The Spearman correlation analysis revealed a direct association between the male sex and the abundance of Pasteurellales microbes, while the age of the patients was directly correlated with Enterobacteriales, Enterobacteriaceae, Flavobacterium, *Parabacteroides distasonis*, and *Bacteroides ovatus*, and inversely correlated to Erysipelotrichi, Coriobacteriales, Erysipelotrichales, Coriobacteriaceae, Lactobacillaceae, *Collinsella*, and *Collinsella aerofaciens*. The disease duration result was positively associated with the species *Bacteroides caccae*, while it was negatively correlated with *Parabacteroides merdae*. Interestingly, a direct correlation between DAS and Euryarchaeota, Gammaproteobacteria, Pasteurellales, and *Anaerobranca zavarzinii* was found, while Erysipelotrichi, Erysipelotrichales, Coriobacteriales, Coriobacteriaceae, Lactobacillaceae, *Collinsella*, *Bacteroides rodentium*, and *Collinsella aerofaciens* were inversely associated with this score. A certain number of correlations were shared by RF and ACPA positivity. In detail, both factors were positively associated with *Roseburia* and negatively with Bacilli, Lactobacillales, and *Streptococcus vestibularis.* In addition, ACPA positivity was indirectly correlated with Streptococcaceae, Erysipelotrichaceae, *Streptococcus*, *Bacteroides xylanisolvens*, and *Lachnospira pectinoschiza.* Direct correlations between ESR and Enterobacteriales, *Roseburia faecis*, and *Streptococcus parasanguinis*, and between CRP and *Parabacteroides distasonis* emerged from the analysis. Regarding lifestyle factors, a varied and well-balanced diet was inversely associated with Pasteurellales, Paraprevotellaceae, *Paraprevotella*, *Blautia*, *Blautia coccoides*, and *Bacteroides eggerthi*, while smoking was positively correlated with Betaproteobacteria, Burkholderiales, Pasteurellales, Lachnospiraceae, Alcaligenaceae, *Roseburia*, *Lachnospira*, *Sutterella*, *Lachnospira pectinoschiza*, *Bacteroides denticanum*, and *Sutterella wadsworthensis*.

Additionally, since the variables affecting the microbiota composition in RA are numerous and can be interconnected, a multivariate approach was followed for a more complete understanding of these interactions. This analysis revealed that independent factors affecting the disease were DAS and Euryarchaeota at the phylum level, DAS and Erysipelotrichi at the class level, and only DAS at the order level. On the contrary, no independent factors were found to correlate with the disease at the family, genus, and species levels (Supplementary Material).

## 3. Discussion

In our pilot study, we applied 16S analysis to characterize the gut microbiota of RA patients, with a particular interest in the effect of synthetic and biotechnological therapies on its composition. Any significant difference in the Shannon index and in the number of species between healthy and diseased subjects was observed in our study population. No change was observed in richness and diversity when patients were classified on the basis of the treatment they received. 

A comparison of the gut microbial populations between healthy and affected individuals pointed out the role of Bacilli in the pathogenesis of RA. Our data are in line with previous studies, in which the related taxa of Lactobacillaceae and *Lactobacillus* were significantly more abundant in RA animal models and patients than in controls. A study by Liu et al. [18] demonstrated that the quantity and variety of lactobacilli in RA patients was higher than in HCs. A few years later, the same authors described an increase of the Lactobacillaceae family and the *Lactobacillus* genus in mice susceptible to developing collagen-induced arthritis, with respect to mice resistance, thus suggesting these bacteria as a predisposing factor of the disease [19]. Moreover, Zhang et al. observed an over-representation of *Lactobacillus salivarius* in the gut and mouth of RA patients, with the highest levels found in people most severely affected [12]. Bacilli (especially *Lactobacillus*) are generally regarded as friendly bacteria for the host; as such, they are among the most commonly used probiotics. Indeed, the administration of *L. casei* and *L. delbrueckii* was shown to alleviate RA symptoms in experimental models [19,20,21,22]; on the other hand, *L. rhamnosus* GG and *L. reuteri* administration failed to ameliorate the disease in patients [23,24,25] suggesting that different *Lactobacillus* species may act differently on RA. 

The decreased abundance of *Faecalibacterium* found in our analysis is in agreement with previous studies carried out on RA [26,27] and other inflammatory conditions [28,29,30]. The bacteria belonging to this genus are well-known butyrate producers, help in maintaining the integrity and health of the gut epithelial barrier, and exhibit anti-inflammatory properties [28,29,30]; thus, their decrease may contribute to the onset of an inflammatory status. No previous association of *Blautia coccoides* (which was depleted in our naïve group) with RA was reported; nonetheless, it was shown that enriching the gut microbiota of systemic lupus erythematosus patients with *Blautia coccoides*, together with *Ruminococcus obeum* and *Bifidobacterium bifidum*, improved the inflammatory status by inducing the production of the immunosuppressive regulatory T cells (Tregs) [31].

As reported by the latest EULAR recommendations, MTX should be considered the first immunosuppressive treatment strategy in patients with RA; in the case of an inadequate response, TNF-α inhibitors or other biotechnological therapies should be started [16]. Zhang et al. previously reported that MTX can affect microbiota composition, partly reversing disease-related dysbiosis [12]. As of now, data on the effect of microbiota in the outcome of patients receiving immunosuppressive biotechnological therapies are limited and only refer to IBD patients. For this reason, we next characterized the gut microbiota of RA patients on different immunosuppressant treatment strategies (ETN, MTX, or ETN plus MTX) and compared it with that of treatment-naïve patients. 

Interestingly, significant changes were found in patients receiving ETN. The phylum of Cyanobacteria and its cognate Nostocophycideae and Nostocales were enriched in the ETN group. Little is known about the role of these microorganisms in health and disease; nevertheless, Cyanobacteria produce secondary metabolites with multiple bioactivities, including anti-inflammatory and immunosuppressant activities [32] which may benefit RA patients. Also, the drop in Deltaproteobacteria caused by ETN could be potentially beneficial if we consider that these microorganisms were found enriched in patients suffering from ulcerative colitis [33] and that Proteobacteria in general are abundant in both intestinal and extra-intestinal inflammatory diseases [34]. Moreover, we observed a decrease in Clostridiaceae upon ETN treatment which could be potentially beneficial since these bacteria were previously found enriched in patients with RA and IBD-associated arthropathy [35]. In patients treated with MTX, our analysis revealed a significant decrease in Enterobacteriales, whose lipopolysaccharides may contribute to inflammation [36], and which were associated with increased intestinal permeability [37], a condition that can be found in RA patients [38].

The results discussed so far further support the evidence of a link between gut microbiota and RA, and show, for the first time, that anti-TNF-α therapy can have a beneficial impact on the microbiota composition. 

With the aim of establishing any association between microbial taxa and clinical parameters of the disease, correlation analyses were performed grouping all forty-two RA patients. Among the most remarkable results, the pro-inflammatory Gammaproteobacteria and its cognate order of Pasteurellales displayed a direct correlation with disease activity (DAS-28), while the order of Enterobacteriales showed a positive association with ESR. Interestingly, the phylum of Euryarchaeota was directly correlated with DAS and emerged as an independent risk factor in RA when a multivariate analysis was performed. The function of these microorganisms in humans is still poorly explored, but their increase was observed in another autoimmune disorder, i.e., multiple sclerosis [39]. 

A positive association between *B. caccae* and disease duration was also found. A membrane protein of *B. caccae*, namely outer membrane protein W (OmpW), was described as a target of the immune response associated with IBD. Intriguingly, this protein is structurally related to a protein of *P. gingivalis* [40], against which increased autoantibody production was discovered in RA [41]. 

In addition to the clinical characteristics, some demographic and lifestyle factors of patients were taken into account. Enterobacteriales and Enterobacteriaceae (belonging to Gammaproteobacteria) were found to be associated with increasing age, while a number of taxa belonging to Betaproteobacteria (Alcaligenaceae, Burkholderiales, *Sutterella*, and *S. wadsworthensis*) were positively correlated with the habit of smoking. 

When compared to previous reports, this study presents the advantage of having evaluated and compared, for the first time, the effects induced by synthetic and anti-TNF-α agents, in monotherapy and combination therapy, on gut dysbiosis in RA patients.

A limit to the current study is that the cross-sectional design can allow us to assess the presence of gut dysbiosis in RA patients who are treatment-naïve, under ETN, and/or MTX therapy; however, it does not give information on the predictive value of these changes in relation to RA clinical outcome and structural progression. 

Overall, the current study revealed that RA is characterized by gut dysbiosis, some of which is associated to the inflammatory status of the disease, suggesting that the microbiota may play an important role in the promotion and clinical course of RA. Moreover, the partial restoration of a beneficial microbiota induced mainly by the anti-TNF-α ETN can contribute to the clinical efficacy of this agent. A deeper understanding of the alterations occurring in the gut microbiota of patients on different therapeutic regimens could help set up individualized and supportive therapeutic strategies providing patients with more effective and safe care. 

## 4. Materials and Methods

### 4.1. Study Population

Forty-two RA patients, according to the European League Against Rheumatism (EULAR)/American College of Rheumatology (ACR) classification criteria [42] were recruited at the outpatient Division of Immunology and Rheumatology, S. Andrea Hospital, Sapienza University of Rome. Patients were divided into four groups according to current therapy: 11 patients were naïve to immunosuppressants, 11 patients were receiving MTX, 10 patients were receiving ETN, and 10 patients were receiving ETN plus MTX. Ten healthy subjects were used as controls (HCs). 

The study was conducted according to the ethical guidelines of the 1975 Declaration of Helsinki. An informed consent was obtained by all the patients and the study was approved by the local ethical Committee (43/2013). All patients were receiving current therapy for at least three months and had to be naïve to other biotechnological drugs. Steroids and non-steroidal anti-inflammatory drugs (NSAIDs) had to be stopped at least seven days before the exams. 

Any patients or HCs on antibiotics, consuming probiotics, or having a known history of inflammatory bowel disease or other autoimmune diseases were excluded.

DAS-28, ESR, CRP, RF, ACPA, and dietary and smoking habits were assessed the same day of stool sample collection.

Patients’ clinical data are described in Table 1.

### 4.2. Sample Collection and DNA Extraction

Each participant collected a fresh stool sample in a collection tube filled with a DNA stabilization buffer (Canvax Biotech, Voden Medical Instruments, Meda, Italy). Then, 250 µL of each sample was processed for microbial DNA extraction using the QIAamp DNA Stool Mini Kit (Qiagen, Milan, Italy) according to the manufacturer’s protocol. DNA concentration and purity were assessed using a NanoDrop spectrophotometer (Thermo Scientific, Meda, Italy).

### 4.3. Next-Generation Sequencing of Bacterial 16S Ribosomal RNA Gene 

The Illumina 16S Metagenomic Sequencing Library Preparation instructions were followed for high-throughput sequencing. Firstly, 12.5 ng of each DNA extract was employed for the amplification of the V3–V4 hypervariable regions of the bacterial 16S ribosomal RNA (rRNA) gene, using the following primers with Illumina adapters (underlined): forward primer: 5′-TCGTCGGCAGCGTCAGATGTGTATAAGAGACAGCCTACGGGNGGCWGCAG, reverse primer: 5′-GTCTCGTGGGCTCGGAGATGTGTATAAGAGACAGGACTACHVGGGTATCTAATCC, selected from Klindworth et al. [43]. The amplification reaction was carried out in the presence of the 2× KAPA HiFi HotStart Ready Mix (Roche, Milan, Italy) under the following conditions: initial denaturation at 95 °C for 3 min, followed by 25 cycles of denaturation at 95 °C for 30 s, primer annealing at 55 °C for 30 s, extension at 72 °C for 30 s, with a final elongation at 72 °C for 5 min. PCR amplicons were then purified by means of Agencourt AMPure XP beads (Beckman Coulter, Milan, Italy). The purified DNA products were then subjected to a further PCR to attach dual Illumina indices (Nextera XT Index Kit, Illumina Inc., San Diego, CA, USA) necessary for multiplexing. The reaction was performed under the following conditions: initial denaturation at 95 °C for 3 min, followed by eight cycles of denaturation at 95 °C for 30 s, primer annealing at 55 °C for 30 s, extension at 72 °C for 30 s, with a final elongation at 72 °C for 5 min. Following a further PCR purification, the eluted DNA products were quantified using the Qubit dsDNA BR Kit assay, diluted to a concentration of 4 nM, and pooled in equal proportion into a single library. Paired-end sequencing (2 × 300 cycles) was carried out on an Illumina MiSeq device (Illumina Inc.) according to the manufacturer’s instructions. Sequences were demultiplexed based on index sequences, and FASTQ files were generated. 

### 4.4. Bioinformatic Analysis

Sequence data were analyzed using the 16S Metagenomics App provided by BaseSpace software (version 1.0.1, Illumina Inc.) which performs taxonomic classification based on the Greengenes database (available online: http://greengenes.secondgenome.com/downloads/database/13_5). For each sample, the relative abundance of the top eight taxonomic classifications at each level (from phylum to species) was considered for statistical analysis. Moreover, the software calculated the Shannon index (α-diversity) and the number of species (richness) found in each sample.

### 4.5. Statistical Analysis

Continuous variables are expressed as means ± standard deviation (SD). Two-group comparisons were calculated using the Student’s *t*-test. Correlation analyses were performed using Spearman rank correlation. Multiple logistic regression analyses with backward variable selection were applied to assess independent correlates of subject status (healthy/pathological). All statistical tests were two-tailed and *p* < 0.05 was considered statistically significant. Statistical analyses were performed by using the R software, version 3.1.0 (10 April 2014)-B Spring Dance Copyright © 2014 The R Foundation for Statistical Computing.

## Figures and Tables

**Table 1:** Main demographic, clinical, and serologic data of the 42 rheumatoid arthritis (RA) patients.

Patient Characteristics	Naïve	ETN	MTX	ETN + MTX	*p*-Value
**Male *n* (%)**	1 (9)	1 (10)	2 (18)	2 (20)	ns
**Age**	55.7	59.8	62.3	64.6	ns
**Varied and balanced diet *n* (%)**	10 (91)	9 (90)	10 (91)	9 (90)	ns
**Smokers *n* (%)**	1 (9)	3 (30)	1 (9)	1 (10)	ns
**Disease Duration (years)**	6.4	14.8	11.2	19.9	0.007 * 0.07 0.002 0.04
**DAS-28**	4.3	3.9	4	3.7	ns
**RF pos *n* (%)**	8 (73)	6 (60)	8 (73)	6 (60)	ns
**ACPA pos *n* (%)**	8 (73)	6 (60)	8 (73)	6 (60)	ns
**ESR (mm/h)**	27.4	28.3	22.4	22.6	ns
**CRP (mg/L)**	5.7	6.5	5.1	6.4	ns

RA = Rheumatoid arthritis; Naïve = patients naïve to immunosuppressants; ETN = etanercept; MTX = methotrexate; ACPA = anti-citrullinated peptide antibodies; RF = rheumatoid factor; ESR = erythrocyte sedimentation rate; CRP = C-reactive protein; DAS-28 = disease activity score on 28 joints. Data are expressed as means. * Naïve vs. ETN; naïve vs. MTX; naïve vs. ETN + MTX; MTX vs. ETN + MTX. ns = not significant.

## Supplementary Materials

Supplementary materials can be found at http://www.mdpi.com/1422-0067/19/10/2938/s1.
