# Supplementary material for: Analysis of Gut Microbiota in Rheumatoid Arthritis Patients: Disease-Related Dysbiosis and Modifications Induced by Etanercept
Source: Int J Mol Sci. 2018 Sep 27;19(10):2938. doi: 10.3390/ijms19102938 (PMC6213034; doi:10.3390/ijms19102938)
Supplement: Supplementary file 1 [file ijms-19-02938-s001.pdf]

Supplementary Materials

Phylum

	Coefficient	*p*-Value
Age	−0.0025	0.4596
Sex	−0.1921	0.0659
Smoke	−0.1013	0.2374
Diet	−0.0222	0.8593
RF	0.1616	0.1977
ACPA	−0.0522	0.7024
ESR	−0.0016	0.6642
RCP	0.0069	0.2604
DAS	0.1623	0.0003
Disease duration	0.0012	0.7626
Actinobacteria	−0.2893	0.2939
Bacteroidetes	−0.2985	0.2795
Caldithrix	8.7069	0.7135
Chloroflexi	5.3327	0.3057
Cyanobacteria	−0.2817	0.3663
Euryarchaeota	−2.094	0.0241
Firmicutes	−0.299	0.2780
Fusobacteria	0.8659	0.6134
Nitrospirae	0.3769	0.7133
Proteobacteria	−0.3390	0.2279
Synergistetes	0.1822	0.7676
Tenericutes	−1.0269	0.0563
Thermotogae	−2.980	0.1709
Unclassified	−0.1288	0.6348
Verrucobacteria	−0.2959	0.2944

Class

	Coefficient	*p*-Value
Age	0.0022	0.4668
Sex	−0.0864	0.3293
Smoke	−0.0917	0.2823
Diet	0.0202	0.859
RF	0.1773	0.1435
ACPA	−0.1500	0.9489
ESR	−0.0053	0.0797
RCP	0.004	0.4528
DAS	0.1423	0.0002
Disease duration	0.0011	0.7727
Alphaproteobacteria	−0.004	0.9489
Bacilli	0.005	0.7099
Bacteroidia	−0.0032	0.7372
Betaproteobacteria	0.0302	0.4625
Clostridia	−0.0067	0.5940
Deltaproteobacteria	−0.1088	0.1673
Erysipelotrichi	0.0735	0.0078
Flavobacteriia	−0.0259	0.2792
Gammaproteobacteria	−0.0244	0.1847
Mollicutes	−01725	0.5747
Nostocophycideae	0.0076	0.8282
Sphingobacteriia	0.0221	0.6312
Synergistia	0.2349	0.5868
Unclassified	0.0053	0.9332
Verrucomicrobiae	−0.0066	0.7948

Order

	Coefficient	*p*-Value
Age	0.0044	0.2357
Sex	−0.1912	0.1197
Smoke	−0.0603	0.5435
Diet	0.0523	0.7157
RF	0.1164	0.4194
ACPA	−0.0537	0.7794
ESR	−0.0040	0.2554
RCP	0.0061	0.3272
DAS	0.1282	0.0053
Disease duration	0.0027	0.5500
Acholeplasmatales	−0.0356	0.9164
Aeromonadales	0.2389	0.9936
Bacteroidales	0.0151	0.6954
Bifidobacteriales	0.0216	0.6023
Burkholeriales	0.0933	0.1622
Clostridiales	0.0142	0.7151
Coriobacteriales	0.0369	0.4086
Desulfovibrionales	−0.1829	0.2763
Desulfuromonadales	0.2452	0.5124
Enterobacteriales	0.0456	0.4523
Erysipelotrichales	0.0388	0.3794
Flavobacteriales	−0.0284	0.5464
Lactobacillales	0.0067	0.8660
Nostocales	0.0796	0.1617
Pasteurellales	0.0608	0.6430
Rhodospirillales	N/A	N/A
Sphingobacteriales	−0.0237	0.7567
Synergistales	0.4927	0.5487
Turicibacterales	−0.5280	0.5094
Unclassified	0.0541	0.7350
Verrucomicrobiales	0.0375	0.3819

Family **Genus**

	Coefficient	*p*-Value		Coefficient	*p*-Value
Age	9.933×10^{-2}	0.5716	Age	0.0077	0.3047
Sex	-5.220×10^{-2}	0.6618	Sex	−0.0925	0.5727
Smoke	-8.745×10^{-2}	0.5695	Smoke	0.0604	0.7578
Diet	-6.821×10^{-2}	0.7412	Diet	0.1647	0.5269
RF	1.823×10^{-1}	0.3757	RF	0.0299	0.8828
ACPA	-1.928×10^{-2}	0.9361	ACPA	0.2291	0.3384
ESR	-7.771×10^{-3}	0.3027	ESR	−0.0063	0.3118
RCP	-6.780×10^{-4}	0.9463	RCP	−0.0033	0.7606
DAS	1.414×10^{-1}	0.0802	DAS	0.0686	0.3954
Disease duration	1.142×10^{-2}	0.2754	Disease duration	0.0067	0.4568
Alcaligenaceae	3.121×10^{-2}	0.6570	*Akkermansia*	0.0358	0.4671
Anaerobrancaceae	-1.850×10^{-1}	0.3572	*Alkaliphilus*	−0.0341	0.5263
Bacteroidaceae	9.793×10^{-5}	0.9935	*Bacteroides*	0.0008	0.9538
Bifidobacteriaceae	2.582×10^{-4}	0.9881	*Bifidobacterium*	0.0193	0.5458
Clostridiaceae	8.937×10^{-4}	0.9759	*Blautia*	0.0187	0.5637
Coriobacteriaceae	2.533×10^{-2}	0.3793	*Clostridium*	0.0561	0.3078
Desulfuvibrionaceae	2.091×10^{-2}	0.9707	*Collinsella*	0.0360	0.3291
Enterobacteriaceae	1.726×10^{-2}	0.3941	*Coprococcus*	−0.0075	0.9335
Enterococcaceae	-5.809×10^{-2}	0.6918	*Dialister*	0.0386	0.4020
Erysipelotrichaceae	2.815×10^{-2}	0.6522	*Dysgomomonas*	−0.0274	0.7381
Flavobacteriaceae	-2.575×10^{-2}	0.4014	*Eggerthella*	−0.0009	0.9982
Lachnospiraceae	-6.152×10^{-3}	0.6461	*Eubacterium*	0.0309	0.7122
Lactobacillaceae	1.906×10^{-2}	0.8853	*Faecalibacterium*	−0.0033	0.8392
Nostocaceae	3.277×10^{-2}	0.5624	*Flavobacterium*	−0.0334	0.2203
Paraprevotellaceae	-2.045×10^{-2}	0.6590	*Lachnobacterium*	−0.0813	0.8828
Peptostreptococcaceae	7.441×10^{-3}	0.8518	*Lachnospira*	0.0240	0.6603
Porhpyromonadaceae	-1.1241×10^{-2}	0.6769	*Magamonas*	−0.0454	0.6647
Prevotellaceae	-2.859×10^{-3}	0.8333	*Mitsuokella*	0.0021	0.9772
Ruminococcaceae	-9.149×10^{-3}	0.5072	*Oscillospira*	0.0078	0.8501
Sphingobacteriaceae	-1.142×10^{-3}	0.9906	*Parabacteroides*	0.0101	0.8517
Streptococcaceae	2.562×10^{-3}	0.9016	*Paraprevotella*	−0.1424	0.5930
Succinivibrionaceae	-3.360×10^{-2}	0.3649	*Phascolarctobacterium*	−0.0021	0.9791
Unclassified	3.664×10^{-3}	0.9235	*Prevotella*	0.0058	0.7540
Veillonellaceae	-4.780×10^{-3}	0.8461	*Roseburia*	−0.0045	0.8850
Verrucomicrobiaceae	-1.699×10^{-2}	0.6704	*Ruminococcus*	0.0053	0.8009
			Streptococcus	0.0225	0.2470
			Succinivibrio	−0.0231	0.4953
			Sutterella	−0.0692	0.5600
			Tetragenococcus	−0.0131	0.9247
			Unclassified	0.0211	0.6159
			Veillonella	−0.0117	0.8991

Species

	Coefficient	*p*-Value
Age	0.0376	N/A
Sex	N/A	N/A
Smoke	N/A	N/A
Diet	0.0523	N/A
RF	2.0596	N/A
ACPA	−3.3820	N/A
ESR	N/A	N/A
RCP	N/A	N/A
DAS	0.4464	N/A
Disease duration	−0.0507	N/A
Akkermansia muciniphila	0.1180	N/A
Alkaliphilus crononatoxidans	−0.0551	N/A
Alkaliphilus peptidifermentans	−05250	N/A
Anaerobranca zavarzinii	0.0508	N/A
Bacteroides caccae	0.5154	N/A
Bacteroides clarus	−0.7977	N/A
Bacteroides coprocola	0.1291	N/A
Bacteroides coprophilus	−0.4894	N/A
Bacteroides denticanum	0.6316	N/A
Bacteroides dorei	0.2492	N/A
Bacteroides eggerthi	0.2596	N/A
Bacteroides fluxus	−0.7791	N/A
Bacteroides fragilis	−0.8098	N/A
Bacteroides massiliensis	0.2400	N/A
Bacteroides ovatus	0.0042	N/A
Bacteroides plebeius	0.0012	N/A
Bacteroides rodentium	0.1631	N/A
Bacteroides salanitronis	0.5717	N/A
Bacteroides stercoris	−0.2675	N/A
Bacteroides thetaiotaomicron	1.5040	N/A
Bacteroides uniformis	0.1386	N/A
Bacteroides vulgatus	0.0377	N/A
Bacteroides xylanisolvens	0.3099	N/A
Balutia coccoides	−0.3024	N/A
Bifidobacterium adolescentis	0.1308	N/A
Bifidobacterium longum	0.0819	N/A
Bifidobacterium stercoris	0.1522	N/A
Blautia hansenii	0.0126	N/A
Caloramator mitchellensis	−0.2299	N/A
Catenibacterium mitsuokai	−0.1411	N/A
Collinsella aerofaciens	0.0937	N/A
Coprococcus eutactus	3.3342	N/A
Dialister invisus	−0.0950	N/A
Dysgomonas wimpennyi	0.0489	N/A
Erysipelothrix inopinata	1.3249	N/A
Erysipelothrix muris	N/A	N/A
Eubacterium biforme	0.1793	N/A
Faecalibacterium prausnitzii	0.1913	N/A
Lachnospira pectinoschiza	−0.4873	N/A
Megamonas funiformis	N/A	N/A
Megasphera elsdenii	−10.9889	N/A
Mitsuokella multacida	4.6313	N/A
Negativicoccus succinivorans	−0.2108	N/A
Novispirillum peregrinum	−2.9861	N/A
Oscillospira eae	−0.4399	N/A
Parabacteroides distasonis	0.0190	N/A
Parabacteroides merdae	−0.1958	N/A
Paraprevotella xylaniphila	N/A	N/A
Pedobacter kwangyangensis	N/A	N/A
Phascolarctobacterium succinatutens	N/A	N/A
Prevotella copri	N/A	N/A
Prevotella stercorea	N/A	N/A
Roseburia faecis	N/A	N/A
Ruminococcus bromii	N/A	N/A
Ruminococcus gnavus	N/A	N/A
Streptococcus parasanguinis	N/A	N/A
Streptococcus thermophilus	N/A	N/A

Streptococcus vestibularis	N/A	N/A
Succiniclasticum ruminis	N/A	N/A
Sutterella wadswothensis	N/A	N/A
Tetragenococcus halophilus	N/A	N/A
Unclassified	N/A	N/A
Veillonella dispar	N/A	N/A
